# Assessment of the Effect of Seed Infection with *Ascochyta pisi* on Pea in Western Canada

**DOI:** 10.3389/fpls.2017.00933

**Published:** 2017-06-12

**Authors:** Nimllash T. Sivachandra Kumar, Sabine Banniza

**Affiliations:** Crop Development Centre, Department of Plant Sciences, University of Saskatchewan, SaskatoonSK, Canada

**Keywords:** *Peyronellaea pinodes*, *Mycosphaerella pinodes*, ascochyta blight, seed components, seed-to-seedling transmission

## Abstract

The role of seed infection with *Ascochyta pisi* using naturally infected seeds with an incidence from 0.5 to 14.5% was studied in field pea experiments in western Canada at locations with historically low inoculum pressure. A significant effect of *A. pisi* seed infection on the emergence of seedlings was observed in one experiment and when all data were pooled, but emergence was only reduced minimally, and symptoms of *A. pisi* on the aerial parts of the seedlings were rarely observed. The level of seed infection at planting had no impact on *A. pisi* disease severity on mature plants, on seed yield and size, or on the incidence of *A. pisi* infection of harvested seeds although *A. pisi* was the dominant species recovered from seeds. Results suggest that the disease did not progress significantly from seeds to seedlings, hence did not contribute to infection of aerial parts of the plants, and therefore infected seeds cannot be regarded as a source of inoculum in the epidemiology of this pathogen under western Canadian growing conditions. Assessing seed components of seeds with varying levels of *A. pisi* infection and seed staining revealed that the pathogen was present in all components of the seed, regardless of the severity of seed staining. This indicates that infected seeds may be an important way for the pathogen to survive in nature.

## Introduction

Ascochyta blight, also referred to as the ascochyta blight complex, is one of the major diseases affecting field pea production and can be caused by several pathogens with anamorphs in the genus *Ascochyta* (Tivoli and Banniza, 2007). Worldwide, *Peyronellaea pinodes* (syn. *Mycosphaerella pinodes*), *Ascochyta pisi*, and *Phoma pinodella* have been associated with this disease. In Australia other species of *Phoma* including *Phoma koolunga* (Davidson et al., 2009), *Phoma herbarum* (Li et al., 2011), and *Phoma glomerata* (Tran et al., 2014) were also shown to be pathogenic on pea and have been associated with ascochyta blight. Among the causal agents of ascochyta blight, *P. pinodes* is considered most damaging with yield losses of 28–88% depending on environmental conditions (Bretag et al., 1995a; Tivoli et al., 1996; Xue et al., 1997; Garry et al., 1998). Symptoms of *P. pinodes* and *Phoma pinodella* are very similar with brown to purplish lesions of irregular shape and without a distinct margin (Jones, 1927). *A. pisi*, in contrast has light brown lesions with a distinct darker brown margin. Pycnidia are easily visible in mature lesions of *A. pisi*, but not in those of the other two species.

Infection of pea seed is one of the major survival mechanisms of *Ascochyta* spp. and an important way of transmission into previously uninfected areas, but for some species can also represent a major source of inoculum for the developing crop (Tivoli and Banniza, 2007). Infection reduces seed germination, and seedlings that do develop from infected seeds may be diseased resulting in poor plant development and stands (Jones, 1927; Maude, 1966; Moussart et al., 1998). Higher severity of seed staining could be correlated with deeper penetration of *P. pinodes* into the seed, which in turn reduced emergence rates (Moussart et al., 1998). Under controlled conditions, seed-to-seedling transmission was up to 100% for *P. pinodes* (Xue, 2000) and 40% for *A. pisi* (Maude, 1966).

The impact of seed-borne inoculum is influenced by factors including rainfall and temperature, and areas with low rainfall often produce disease-free seeds in the field (Bathgate et al., 1989; Bretag et al., 1995b). Surface sterilization of pea seeds results in a reduction of seed infection with *P. pinodes* by 60%, indicating that the pathogen may be mostly carried on the seed coat (Bathgate et al., 1989). Seed infection levels with *P. pinodes* higher than 10% can cause severe economic damage to the crop under conducive environmental conditions (Xue, 2000). Seed-borne infection of other species of the ascochyta blight complex such as *Phoma* spp. has not been identified as very important in initiating epidemics of ascochyta blight in the field. *Ascochyta* spp. can survive on pea seed coats for several years (Bretag et al., 1995b), and for *A. pisi* specifically, it was estimated that the fungus will be eliminated from seed after 5 to 7 years of seed storage in cool and dry conditions (Wallen, 1955).

Until 1961, *A. pisi* was the dominant pathogen recovered from pea seeds in Canada (Wallen et al., 1967a). Incidences of 85% seed infection with *A. pisi*, 27.5% with *P. pinodes* and 10% with *Phoma pinodella* were reported from Canada in the mid-1950s (Skolko et al., 1954). In 1961, the pea variety Century (originally released as Creamette [Gfeller and Wallen, 1961]) was introduced and quickly gained in acreage due to its high level of resistance to *A. pisi*. Simultaneously, *P. pinodes* became the dominant foliar pea pathogen in Canada (Wallen et al., 1967a). In the early 2000s, a resurgence of *A. pisi* was noted in western Canada based on increasing levels of this pathogen on harvested seeds (Morrall et al., 2011). In response to this, experiments were conducted to reassess the impact of seed infection in the epidemiology of *A. pisi*, to evaluate the importance of seed-to-seedling transmission under field conditions, and to determine the nature of seed-borne infection by *A. pisi*. It was hypothesized that pea plants developing from seeds infected with *A. pisi* would be infected with the pathogen and that seed infection would thus promote the development of *A. pisi* infection in the developing crop canopy. It was also hypothesized that low levels of seed coat staining would be indicative of no or a low incidence of embryo infection with *A. pisi* whereas high seed coat staining would be correlated with a high incidence of embryo infection.

## Materials and Methods

### Field Experiments

Seeds of CDC Patrick, a green cotyledon field pea cultivar, were used for this experiment. Two commercial seed lots with an incidence of natural *A. pisi* seed infection of 0.5 and 14.5%, and 0 and 4% *P. pinodes* infection, respectively, confirmed by a commercial seed testing lab, were obtained from a seed grower. Samples were combined to obtain *A. pisi* incidence levels of 0.5, 5, 10, and 14.5%, which were confirmed through seed testing by plating four replicates of 100 seeds per incidence level onto potato dextrose agar (PDA) after 2.5 min surface sterilization in 0.6% NaOCl. Field experiments were established in the Canadian province of Saskatchewan in May at Outlook, Saskatoon, and Milden where levels of *A. pisi* infection had been low in previous years, and experiments were harvested in August. Detailed dates and general agronomic practices are presented in Supplementary Table S1. Experiments were designed as randomized complete block designs with four replicates. Plot size was 1.2 m × 3.7 m with 26 seeds m^-1^ row, or 86 seeds m^-2^ at a row spacing of 30 cm.

During the growing season, plant emergence was assessed by counting the number of seedlings per one meter plant row in four arbitrarily selected rows or row segments of each plot. The severity of symptoms caused by *A. pisi* and *P. pinodes* was assessed at the seedling stage, during flowering, at the podding stage and at maturity using the 0–10 rating scale based on 10% incremental increases in the percentage of disease severity together on leaves, stems and eventually pods. Five arbitrarily selected plants were rated in each plot and data were transformed to percentage disease severity using the class mid points. The averages per plot were calculated for further data analyses.

At harvest, seed yields were determined for each plot, seeds were assessed for thousand seed weight (TSW) and the incidence of seed infection with pathogens.

For seed testing, 100 seeds per plot were surface-sterilized by soaking in 0.6% NaOCl for 3 min with constant agitation, rinsing with sterile distilled water for 2 min, and drying on a sterile distilled paper towel before plating on PDA plates at 10 seeds per 9 cm Petri dish. Seeds were incubated at 20°C for 7 days under continuous fluorescent light on the bench top. Each seed was assessed for infection by *A. pisi*, *P. pinodes*, and other pathogens, and the percentage incidence of infection was recorded per plot for each pathogen.

### Seed Component Study

The same source of CDC Patrick seeds as above with an incidence of *A. pisi* infection of 14.5% was used for the seed component study. Based on the relatively low level of 4% *P. pinodes* infection in this sample, it was assumed that seed coat staining was primarily caused by *A. pisi* infection. The seeds were visually categorized into five categories based on the amount of seed coat staining of individual seeds: 0% (clean seeds without any staining), 1 to 25%; 26 to 50%; 51 to 75%; 76 to 100% of the seed coated stained. The latter also included a small number of underdeveloped and shriveled seeds assumed to be caused by *A. pisi* (**Supplementary Figure S1**). For each category, seven replicates of 50 seeds were soaked in sterile distilled water for 2 h to soften the seed coat. Seeds were dissected into seed coat, cotyledon, and embryo. Seed components were surface-sterilized by soaking in 0.6% NaOCl for 3 min with constant agitation, rinsing with sterile distilled water for 2 min, and drying on a sterile distilled paper towel before being placed on PDA in Petri dishes. Seeds were incubated at 20°C for 7 days under continuous fluorescent light in a bench top incubator. Each Petri dish was assessed for infection and fungal growth was morphologically identified to the species level for *A. pisi* and *P. pinodes*, and to the genus level for other common fungi.

### Data Analysis

All data were analyzed using in SAS (Version 9.4, SAS Institute Inc.). All data were tested for normality and heterogeneity of variances of residuals. Data of emergence, yield, TSW, disease severity and the incidence of *A. pisi* infection were analyzed with the regression procedure where the seed infection level was the regressor. Incidence data for *A. pisi* and *P. pinodes* from the seed component study were analyzed with the mixed model procedure where seed staining categories and seed components were considered fixed effects, whereas replications were considered random effects. Initially, other pathogens detected in seed samples were used as covariates. Final modeling of *A. pisi* data was done with the significant covariate(s) and means were separated by Fisher’s least significant difference test.

## Results

### Field Experiments

**FIGURE 1:**
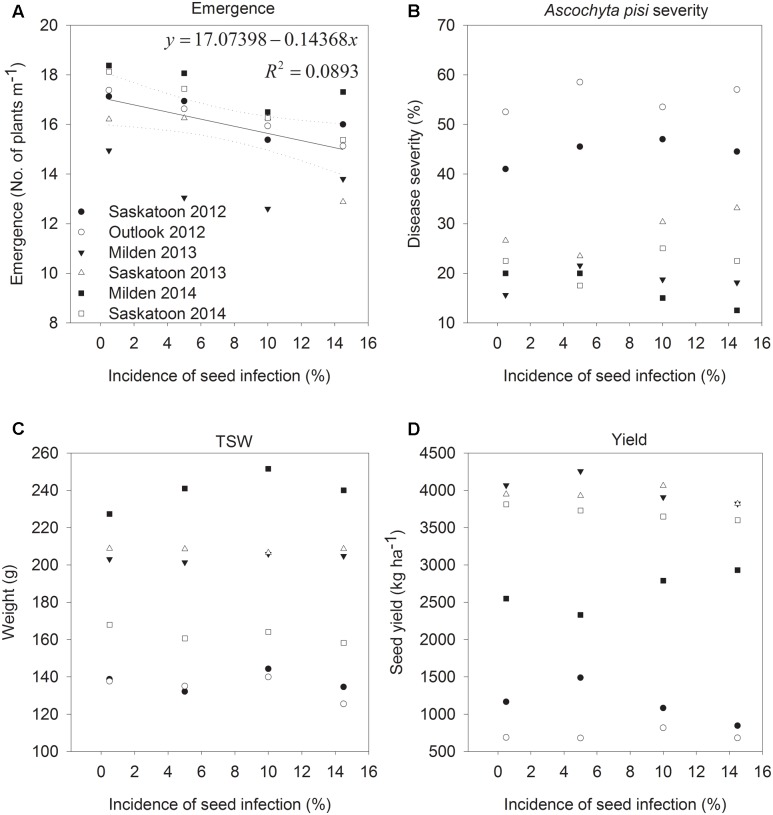
Seedling emergence **(A)**, *Ascochyta pisi* severity on mature plants **(B)**, seed yields **(C)**, and thousand-seed weight (TSW; **D**) of pea cv. CDC Patrick grown from seeds with incidence levels of *A. pisi* infection of 0.5, 5, 10, and 14.5% in field experiments conducted at two locations in 2012 to 2014.

Seedling emergence ranged from 10 to 24 seedlings per meter row in plots, with an overall average of 16 seedlings per meter row. Emergence was lowest at Milden in 2013 and highest at the same location in 2014, which was most likely associated with soil moisture conditions during emergence. Infection of CDC Patrick seeds with *A. pisi* only reduced emergence at Outlook in 2012 (*P* = 0.0306) and when data from all years and locations were pooled (*P* = 0.0031; **Figure 1**). However, in both cases, seed infection only explained a small proportion of the variability in emergence (29% for Outlook 2012, 9% for pooled data), and based on pooled data emergence was reduced by 1 plant m^-1^ row for every 7% increases in the incidence of seed infection.

The average severity of *A. pisi* symptoms on seedlings after emergence was 1% in 2012 and 2013, and 5% in 2014, and many seedlings were disease-free. Overall, disease development on peas was higher at Saskatoon and Outlook in 2012 than in other years and locations because of higher precipitation (359 and 343 mm, respectively, compared to 143 to 234 mm in other years and locations) during the growing season (May to August). Temperatures were similar with maximum deviations among average daily temperatures for each month of 3°C. Seed infection with *A. pisi* had no effect on *A. pisi* development of pea seedlings (data not shown) or plants close to maturity when average *A. pisi* symptom severity ranged from 17 (Milden 2014) to 55% (Outlook in 2012). The severity of *P. pinodes* ranged from 18 (Saskatoon 2014) to 62% (Saskatoon 2012), and was always higher than *A. pisi* severity, with the exception of Saskatoon in 2014, when the severity of *A. pisi* reached 22%, whereas it was 18% for *P. pinodes* when averaged across all treatments. There were no significant differences in *P. pinodes* severity among treatments in any of the experiments.

Seed infection with *A. pisi* had no effect on seed yields, TSW or the incidence of *A. pisi* infection of harvested pea seeds (**Figure 1**). *A. pisi* infection of harvested seed was close to 0 at Outlook in 2012, but reached an average of 7% at Saskatoon in 2012. The incidence of *P. pinodes* infection ranged from 0.4% at Saskatoon in 2013 to 9% at Milden in 2014, and similar to *A. pisi*, there were no treatment effects. Except for Outlook 2012 and Milden 2014, harvested seeds had more *A. pisi* than *P. pinodes* infection.

### Seed Component Study

**FIGURE 2:**
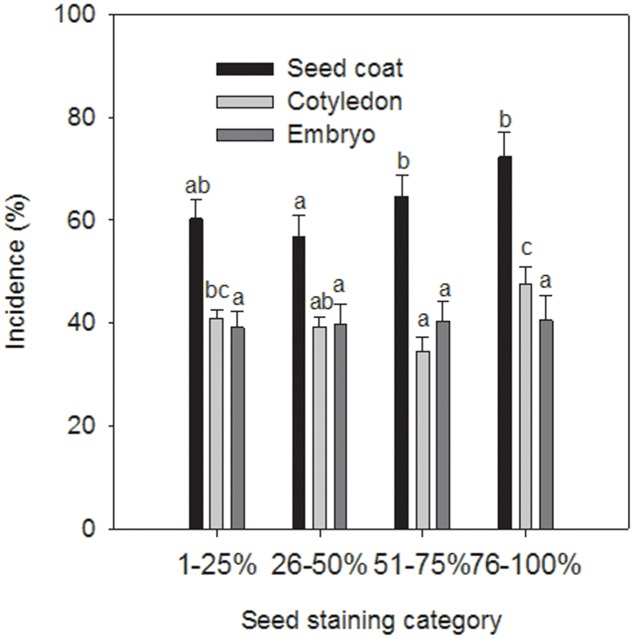
Incidence (%) of *Ascochyta pisi* infection of seed coats, cotyledons, and embryos of commercial pea seeds of cv. CDC Patrick that were separated into four seed coat staining categories. Bars indicate standard errors of the mean. Letters above columns indicate significant differences: columns of each series with a letter in common are not significantly different.

Seed components without staining of the seed coat were not infected with *A. pisi*. Seed coats, cotyledons, and embryos of all other four seed staining categories were infected with *A. pisi*. In addition to *A. pisi*, other organisms, such as *Colletotrichum* spp., *Fusarium* spp., *Alternaria* spp., *Epicoccum* spp., unidentified green molds and bacteria were also identified on the stained seed components (**Table 1**). Only incidence data of *Epicoccum* spp. had a significant effect on the model as a co-variate (*P* = 0.0212) and were included in the model. Seed staining category, seed components, and their interaction had significant effects on the incidence of *A. pisi* infection (*P* < 0.0001). Seed staining categories 51–75% and 76–100% had a higher incidence of seed coat infection compared to that in staining category of 26–50%. Seeds staining categories 1–25% and 76–100% had a higher incidence in cotyledon infection compared to staining category 51–75%, whereas there was no difference in the incidence of embryo infection among the seed staining categories (**Figure 2**).

**Table 1:** Incidence levels (%) of *Ascochyta pisi* and other fungi (mean of 3 seed components) on naturally infected seeds of pea cv. CDC Patrick seeds that were separated into four seed coat staining categories.

	Staining category							
	
Pathogens	1–25%		26–50%		51–75%		76–100%	
*Ascochyta pisi*	45.36	(1.24)	43.71	(2.46)	43.50	(2.46)	52.00	(2.78)
*Peyronellaea pinodes*	0.14	(0.14)	0.14	(0.14)	2.14	(0.70)	6.29	(1.34)
*Alternaria* spp.	3.71	(0.97)	7.57	(1.34)	10.00	(1.46)	25.14	(2.51)
*Colletotrichum* spp.	0.71	(0.42)	1.14	(0.86)	3.57	(1.13)	7.14	(1.20)
*Stemphylium* spp.	0.57	(0.20)	3.86	(0.94)	3.43	(1.02)	3.71	(1.02)
*Epicoccum* spp.	0.14	(0.14)	0.86	(0.34)	0.29	(0.18)	1.00	(0.44)
Green mold	2.86	(0.80)	2.43	(0.53)	9.86	(1.74)	14.29	(1.71)
Bacteria	0.57	(0.57)	0.14	(0.14)	1.57	(0.53)	1.71	(0.81)


*Numbers in brackets represent standard errors of the mean.*

## Discussion

Pea seedling emergence was slightly, but statistically significantly affected by the incidence of *A. pisi* infection of seeds. Based on the regression model here, an increase in the incidence by 7% *A. pisi* infection in seeds is predicted to reduce seedling emergence by 1 plant m^-1^ representing 4% in our experiment with 26 plants m^-1^. This indicates that even an incidence of 14.5% of seed infection, the highest infection level assessed here, will only have a minor impact on plant stands. A much more significant impact of *A. pisi* seed infection on emergence was reported previously by Jones (1927) who found 69 and 76% seedling emergence under field, and 75% under greenhouse conditions from a seed sample with an incidence of *A. pisi* infection of 8%, when compared to emergence of seeds from the same sample treated with organic mercuric dust. In contrast, assessments of seed samples from several years and locations with *A. pisi* infection rates of 10% resulted in seedling emergence of 85% (Wallen, 1955). In that study, samples with 44 and 46% *A. pisi* infections were assessed as well and had emergence rates of 87 and 67%, respectively, supporting observations here that *A. pisi* infection does not have a major impact on emergence, although the confounding impact of organisms other than *A. pisi*, observed in all of these studies, has not been quantified. When comparing these numbers it is important to keep in mind that the earlier reports used pea varieties that are now 60 to more than 100 years old, and were most likely more susceptible to *A. pisi* than modern CDC Patrick. Even though the first highly *A. pisi* resistant pea variety was only released in 1961 (Gfeller and Wallen, 1961), it can be speculated that pea varieties studied by Wallen (1955) may have already had improved resistance compared to those used by Jones (1927) 28 years earlier, as resistance to *A. pisi* will have been a primary breeding objective. A negligible impact of *A. pisi* on pea seedling emergence observed here is in stark contrast to *P. pinodes* where seed infection levels of 24 to 46% resulted in germination rates of 19 to 23% (Xue, 2000), and seeds with more than 50% seed coat staining had a seed-to-seedling transmission of 100% (Moussart et al., 1998).

Precipitation during the growing seasons of 2012 to 2014 at experimental locations was average or above average, so conditions generally were conducive for the initiation of epidemics. Very low levels of seedling infections and no effect of *A. pisi* seed infection on disease severity on the developing plants here indicated that infection of seeds with *A. pisi* used for seeding does not pose a risk for initiating epidemics in the field under western Canadian growing conditions. There was also no effect on seed yield, seed size or the infection levels with this pathogen of harvested seeds. In general, *A. pisi* is considered to be less aggressive than other pathogens, with reported yield losses of 11% compared to 45 and 25% due to *P. pinodes* and *Phoma m.* var *pinodella*, respectively (Wallen, 1965).

In four of the six field experiments seed infection of harvested seeds with *A. pisi* was higher than with *P. pinodes* despite the fact that for three of those four experiments, *P. pinodes* severity on pea plants was higher than *A. pisi* severity. Wallen et al. (1967b) pointed out a natural antagonism between *A. pisi* and *P. pinodes*, and also found that seed-borne infection tends to be higher with *A. pisi* compared to *P. pinodes* (Wallen, 1965). A higher incidence of *A. pisi* infection had been observed for certain seed lots in commercial seed testing labs in recent history as well (Morrall et al., 2011), which had triggered a re-assessment of the importance of *A. pisi* here. When assessing seed components for infection, the embryo of all seeds were infected with *A. pisi* irrespective of the amount of seed staining as long as there was some seed coat staining. This is distinctly different from seed infection by *P. pinodes* where the amount of seed coat staining is positively correlated with the depth of infection into the seed and the frequency of embryo infection (Moussart et al., 1998). For this pathogen, no necrosis on seed components other than on the seed coat was observed for seeds with less than 25% seed staining. Seeds with higher seed coat staining always showed evidence of necrosis caused by *P. pinodes* on the outward facing side of cotyledons, and a gradual increase in the incidence of necrosis on the inward-facing side of cotyledons from 12 to 100% as outer seed coat staining increased from 25 to 100%. Similarly, the incidence of necrosis on embryos increased from 10 to 100% once seed coat staining exceeded 25% and increased to 75 to 100%. This positive correlation between increasing outer seed coat staining and infection of inner seed components suggests that *P. pinodes* infects the more or less immature pod and penetrates from there into the seeds. The relatively high incidence of *A. pisi* in embryos and cotyledons irrespective of the amount of seed coat staining may indicate that *A. pisi* infection already occurs during flowering. The lack of correlation between foliar infection, from which water-splashed conidia could infect flowers, and the incidence or depth of seed infection indicates that airborne ascospores of *A. pisi* rather than water-splashed conidia may infect flowers and seeds, considering that windborne ascospores can be blown in from remote inoculum sources, and ascospore concentration will likely be more equal across a field. Little is known about the life cycle of *A. pisi* whereas that of *P. pinodes* has been well studied. The latter is homothallic and readily produces sexual fruiting structures (pseudothecia) which are thought to overwinter on pea stubble generating airborne ascospores that represent the initial inoculum for the new pea crop in the following season (reviewed in Roger and Tivoli, 1996). Studies in France showed that ascospores of *P. pinodes* are released throughout the growing season, but peak toward its end when large numbers of pseudothecia develop almost exclusively on senescent plant tissue, and mostly on stems of the maturing, increasingly diseased and senescent pea plants.

The teleomorph of the heterothallic species *A. pisi, Didymella pisi*, was only described relatively recently and it was shown that pseudothecia matured within 2 months at a constant temperature of 10°C, but their development ceased at 23°C (Chilvers et al., 2009). Historically, the daily maximum temperature in many parts of the Canadian Prairies exceeds 23°C during the growing season, but the daily average temperature often does not due to cool nights, so depending on the effect of fluctuating temperatures on perithecial development in *A. pisi* the climate may be conducive for ascospore production. To date, no studies have been conducted to determine whether pseudothecia develop under field conditions, nor have there been attempts to trap ascospores of this species. Indeed, such research would be complicated by the fact that *P. pinodes* tends to also be present. Although pseudothecia of *A. pisi* are slightly larger than those of *P. pinodes*, ascospores overlap in size (Punithalingam and Holliday, 1972, Chilvers et al., 2009), hence differentiating sexual structures of both species by microscopying or spore trapping would be highly complicated. Molecular probes readily differentiate between them, but do not allow to determine whether fruiting structures and spores are of sexual or asexual origin. In future, it may be possible to conduct studies of this nature through a combination of sophisticated imaging technology and molecular techniques.

Jones (1927) suggested that *A. pisi* overwinters as mycelium on pea straw after inoculating pea stems with this pathogen and incubating them under natural winter conditions in Wisconsin, United States. However, based on Wallen et al. (1967b) isolation of *A. pisi* from agricultural soil of eastern Canadian fields or from sterilized soil inoculated with spore suspensions of the ascochyta blight pathogens was unsuccessful whereas *P. pinodes* and *Phoma pinodella* were isolated on a regular basis. Incubation studies in sterilized soil each inoculated with one of the three ascochyta blight pathogens and incubated at temperatures ranging from -20 to +30°C revealed that *A. pisi* only survived in the soil for a period of 12 months at +5 and -20°C (Wallen and Jeun, 1968). At -20°C, *P. pinodes* and *Phoma pinodella* survived for that period as well, but with lower recovery rates than *A. pisi*. Both, *P. pinodes* and *Phoma pinodella* also survived up to 12 months in soil incubated at 5 to 25°C and were recovered at high rates, indicating clear temperature optima for *A. pisi*, and *P. pinodes* and *Phoma pinodella*. When sterilized soil was co-inoculated with the ascochyta blight pathogens in all possible pairwise combinations, *P. pinodes* was always recovered at the highest rate. In the presence of *P. pinodes*, *Phoma pinodella* survived for at least 9 months, whereas *A. pisi* was least competitive in the presence of either partner.

Jones (1927) also noted that seedlings developing from infected seed had lesions on the first leaves, so may represent a second source of initial inoculum. Testing commercial seed samples, Maude (1966) only found 40% of seed-to-seedling transmission for *A. pisi* compared to close to 100% for *P. pinodes*, and research here with a modern cultivar of pea revealed rare seed-to-seedling transmission under western Canadian field conditions. Considering that the pathogen is not readily isolated from soil (Wallen et al., 1967b), competes poorly with *Phoma pinodella* and *P. pinodes* in soil and does not, or rarely, produces chlamydospores (Wallen and Jeun, 1968), it can be speculated that infected seeds may play a much more important role for the survival of *A. pisi* than is the case for the other two common ascochyta blight pathogens. This would explain why the incidence of seed infection with *A. pisi* historically, and in some years in recent times, has been higher compared to *P. pinodes*.

## Conclusion

The effect of *A. pisi* infection in seed on emergence was minimal under western Canadian growing conditions, *A. pisi* symptoms on seedlings were rare, and incidence levels of *A. pisi* infection of seed up to 14.5% did not increase the amount of disease on mature plants or harvested seeds. Infection with *A. pisi* of harvested seeds was common across all seed infection categories used for seeding, and staining was significant, so while seed infection up to the incidence level tested here may not impact pea production when the seed is used for seeding, the staining caused by *A. pisi* infection of seeds can result in lower quality of seeds to be sold as food or feed. The common infection of embryos and cotyledons of seeds of all staining categories may be indicative for a more dominant role of the seeds in the survival of *A. pisi* compared to *P. pinodes* that survives well in soil. Whether seed infection is initiated by ascospores during flowering, as speculated here, will only be revealed when more is known about the life cycle of this pathogen.

## Author Contributions

NSK conducted the research experiments as part of his MSc thesis, he contributed to the data analyses and to drafting the manuscript. SB was the principal investigator of this research project, supervised NSK, contributed to data analyses and the drafting of the manuscript.

## Conflict of Interest Statement

The authors declare that the research was conducted in the absence of any commercial or financial relationships that could be construed as a potential conflict of interest.

## Supplementary Material

**FIGURE S1** | Seed staining categories based on the percentage of seed coat staining of commercial CDC Patrick field pea with 14.5% incidence of *Ascochyta pisi* infection due to natural infection. Top right: The category with 76 to 100% seed coat staining also included a small number of shriveled and undeveloped seeds.
