# Supplementary figures and images for: Assessment of the Effect of Seed Infection with Ascochyta pisi on Pea in Western Canada
Source: Front Plant Sci. 2017 Jun 12;8:933. doi: 10.3389/fpls.2017.00933 (PMC5466995; doi:10.3389/fpls.2017.00933)

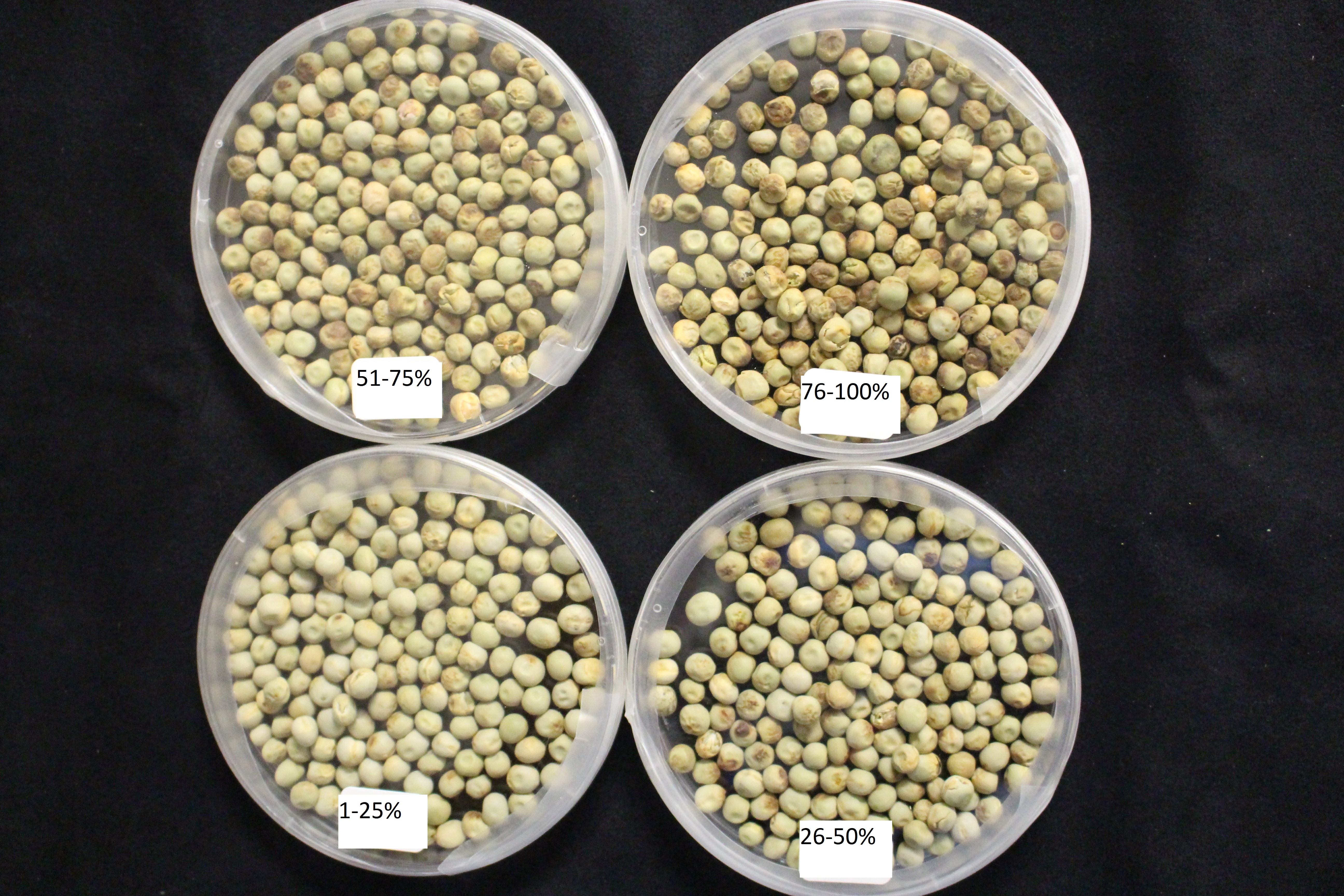

Supplement: Supplementary file 1 [file Image_1.JPEG]
