# Supplementary material for: Assessment of the Effect of Seed Infection with Ascochyta pisi on Pea in Western Canada
Source: Front Plant Sci. 2017 Jun 12;8:933. doi: 10.3389/fpls.2017.00933 (PMC5466995; doi:10.3389/fpls.2017.00933)
Supplement: Supplementary file 2 [file Table_1.pdf]

Table 1. Agronomic practices in field experiments to determine seed-to-seedling transmission of *Ascochyta pisi* in CDC Patrick pea at Outlook, Milden and Saskatoon, Canada, in 2012-2014.

Agronomic practices	2012		2013		2014	
	**Outlook**	**Saskatoon**	**Milden**	**Saskatoon**	**Milden**	**Saskatoon**
**Pre-seeding herbicide (fall application)**	69.19 mL ha^{-1} Pursuit (22.87% imazethapyr) 29.16 kg ha^{-1} Edge (5% ethalfluralin)	69.19 mL ha^{-1} Pursuit (22.87% imazethapyr) 29.16 kg ha^{-1} Edge (5% ethalfluralin)	29.16 kg ha^{-1} Edge (5% ethalfluralin)	29.16 kg ha^{-1} Edge (5% ethalfluralin)	29.16 kg ha^{-1} Edge (5% ethalfluralin)	69.19 mL ha^{-1} Pursuit (22.87% imazethapyr) 29.16 kg ha^{-1} Edge (5% ethalfluralin)
**Seeding**	25 May	30 May	7 May	11 May	10 May	12 May
**Post-seeding herbicide**	**None**	25 June 2.2. L ha^{-1}Basagran forte (480g L^{-1} Bentazon)	11 June 0.59 L ha^{-1} Axial (50 g L^{-1} Pinoxaden) 11 June 185 mL ha^{-1} Centurion (240g L^{-1} Clethodim)	11 June 0.59 L ha^{-1} Axial (50 g L^{-1} Pinoxaden) 11 June 185 mL ha^{-1} Centurion (240g L^{-1} Clethodim)	6 June 0.59 L ha^{-1} Axial (50 g L^{-1} Pinoxaden) 11 June 185 mL ha^{-1} Centurion (240g L^{-1} Clethodim)	6 June 0.59 L ha^{-1} Axial (50 g L^{-1} Pinoxaden) 11 June 185 mL ha^{-1} Centurion (240g L^{-1} Clethodim)
**Emergence count**	21 June	11 July	12 June	14 June	10 June	10 June
**1st Disease rating**	21 June	11 July	12 July	10 July	25 June	26 June
**2nd Disease rating**	26 July	26 July	30 July	13 August	15 July	16 July
**3rd Disease rating**	9 August	13 August	14 August	21 August	30 July	26 July
**Desiccant application**	15 August 2.5 L ha^{-1} Reglone (240 g L^{-1} Diquat)	17 August 2.5 L ha^{-1} Reglone (240 g L^{-1} Diquat)	23 August 2.5 L ha^{-1} Reglone (240 g L^{-1} Diquat)	16 August 2.5 L ha^{-1} Reglone (240 g L^{-1} Diquat)	18 August 2.5 L ha^{-1} Reglone (240 g L^{-1} Diquat)	18 August 2.5 L ha^{-1} Reglone (240 g L^{-1} Diquat)
**Harverst date**	17 August	29 August	30 August	29 August	20 August	25 August
